# The potential of anthocyanin-loaded alginate hydrogel beads for intelligent packaging applications: Stability and sensitivity to volatile amines

**DOI:** 10.1016/j.crfs.2023.100560

**Published:** 2023-08-05

**Authors:** Samira Mohammadalinejhad, Marcin Kurek, Ida-Johanne Jensen, Jørgen Lerfall

**Affiliations:** a Department of Biotechnology and Food Science, NTNU - Norwegian University of Science and Technology, 7491, Trondheim, Norway; b Department of Technique and Food Product Development, Institute of Human Nutrition Sciences, Warsaw University of Life Sciences, 02-776, Warsaw, Poland

**Keywords:** Anthocyanin, Alginate hydrogel bead, pH indicator, Volatile amines, Intelligent packaging

## Abstract

pH indicators have emerged as promising tools for real-time monitoring of product freshness and quality in intelligent food packaging applications. However, ensuring the stability of these indicators is critical for practical use. This study aims to evaluate the stability of anthocyanins-loaded alginate hydrogel beads of varying sizes at different temperatures under accelerated light conditions and relative humidity (RH) levels of 53% and 97% during 21 days of storage. Moreover, their sensitivity to the principal spoilage volatiles of muscle food products such as ammonia (NH_3_), dimethylamine (DMA) and trimethylamine (TMA) was investigated. The half-life of cyanidin-3-glucoside in small hydrogel beads was roughly twice as long as that of the larger beads under accelerated light exposure at 4 °C and they were less likely to undergo noticeable color changes over time. Both sizes of hydrogel beads stored at 97% RH and 4 °C showed color stability over the 21-day period with minimal color variation (|ΔE| ≤ 3). The UV–vis spectra of the purple corn extract exhibited changes across pH 2 to 12, as evidenced by the visible color variations, ranging from pink to green. The limit of detection (LOD) for NH_3_ was 25 ppm for small beads and 15 ppm for large ones. Both types of beads exhibited similar LOD for DMA and TMA, around 48 ppm. This research showed that alginate hydrogel beads containing anthocyanins from purple corn are a viable option for developing intelligent packaging of muscle foods. Furthermore, the use of hydrogel beads of different sizes can be customized to specific muscle foods based on the primary spoilage compound generated during spoilage.

## Highlights

• Anthocyanins-loaded alginate hydrogel beads were introduced as freshness indicators.
• Anthocyanins were loaded into alginate hydrogel beads of different sizes.
• Color stability assessed under light and simulated relative humidity of fresh muscle foods.
• Developed hydrogel-based indicators were sensitive to ammonia, dimethylamine, and trimethylamine.
• Small and large beads were suitable for integration into specific muscle foods.

## Graphical abstract

**Figure:**
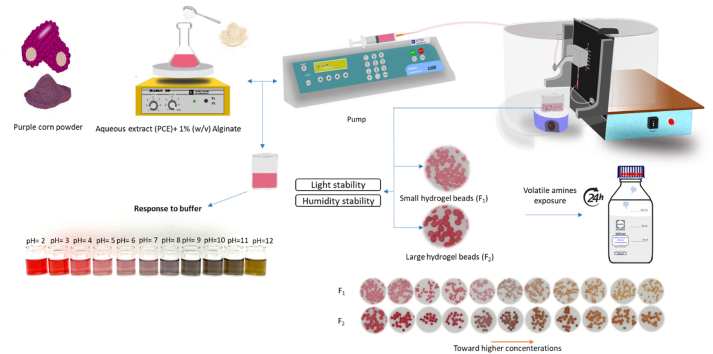

## 1 Introduction

In the European Union (EU), 8.8 million tons of food is wasted annually due to date labeling and misconceptions about how expiry dates are related to food quality (European Commission, 2018). Disposing of a significant amount of safe and edible food highlights the need to develop packaging systems that provide real-time information about the status of the food product in the supply chain.

Intelligent packaging systems are continuously being developed to reveal *in-situ* information about the physical, chemical, and microbiological quality to consumers, retailers, and manufacturers, thereby mitigating food waste (Priyadarshi et al., 2021). These systems are classified into indicators (temperature, leak, and freshness indicators), sensors (chemical and biosensors), and data carriers (radio frequency identification and barcodes). Considering their versatility, simplicity, low cost, and efficiency, freshness indicators appear as promising candidates in intelligent food packaging. A pH indicator is a type of freshness indicator that uses a pH-sensitive dye to provide qualitative information through a colorimetric approach (Kalpana et al., 2019; Mohammadalinejhad et al., 2020). Quality loss in muscle food products is accompanied by an increase in levels of biogenic amines and total volatile basic nitrogen (TVB-N) such as trimethylamine, dimethylamine, and ammonia, as well as microbial metabolites, resulting in pH changes within the headspace (Forghani et al., 2022). The pH indicators undergo a color change during storage that can be correlated with product quality characteristics, such as TVB-N, biogenic amines, pH, and microbial growth (Luo et al., 2022a).

Colorimetric pH indicators commonly comprise a pH-responsive dye and a supporting substrate upon which the dye is immobilized (Mohammadalinejhad et al., 2020). A successful colorimetric pH indicator should meet the following requirements to be applied in the intelligent packaging (Becerril et al., 2021): (i) demonstrate responsive and rapid monitoring of certain or groups of components; (ii) show visually distinguishable color changes that correlate with the quality changes during storage; (iii) be sensitive, stable, cost-effective, and simple to integrate into packaging and (iv) allow real-time tracking of quality without using sophisticated equipment.

Dyes derived from natural sources and food wastes are preferred over chemical counterparts as they are environmentally friendly, renewable, and safe. Natural pigments such as anthocyanins, curcumin, betalain, carotenoids, and quercetin have been used to develop colorimetric pH indicator films (Etxabide et al., 2021; Priyadarshi et al., 2021; Terra et al., 2021). Among them, anthocyanins are the focus of interest owing to their potential biologically active nature and pH-dependent color-changing properties.

Anthocyanins are phenolic phytochemicals that belong to the flavonoids class and are characterized by a C6–C3–C6 basic skeleton. They are typically glycosides and acyl glycosides of anthocyanidins characterized by two benzyl rings (A and B) and a heterocyclic ring (C) (Mohammadalinejhad and Kurek, 2021). One of the most remarkable properties of anthocyanins is their color changes in response to different pH levels because of the resonant structure of the flavylium cation undergoing electron transition by pH variation (Smeriglio et al., 2016). This striking characteristic makes them a suitable candidate to be employed in intelligent packaging to track changes in the pH of food products in real-time (Hashim et al., 2023).

Purple corn (*Zea mays* L.) has been extensively used as a commercial source of anthocyanins and is categorized by the European Union with code E−163 (iv) as a food colorant (Lao and Giusti, 2018). The main bioactive compounds in *purple corn extract (PCE)* are anthocyanins, commonly cyanidin-3-glucoside, cyanidin-3-(6″-malonylglucoside), pelargonidin-3-glucoside, pelargonidin-3-(6″-malonylglucoside), peonidin-3-glucoside and peonidin-3-(6″-malonylglucoside) (Lao and Giusti, 2016). Cyanidin 3-glucoside has been identified as the major anthocyanin in purple corn in several studies (Chen et al., 2018; de Pascual-Teresa et al., 2002; Yang and Zhai, 2010). Furthermore, significant amount of acylated anthocyanins has been reported in it (Jing, 2006; Vidana Gamage et al., 2021). Acylated anthocyanins exhibit higher color stability in aqueous systems than non-acylated ones, which is a critical parameter to consider when choosing indicator colorants (Forghani et al., 2022; Mohammadalinejhad et al., 2020).

The supporting substrate or matrix in which the dye is immobilized is also critical for the sensitivity of the indicator. Up to now, a variety of biopolymers have been used for incorporating anthocyanins, including pectin/sodium alginate/cellulose nanocrystals (Lei et al., 2023), chitosan/gelatin (Lu et al., 2022), alginate/methylcellulose (Sutthasupa et al., 2021), pectin (Guo et al., 2022), gellan gum (Wu et al., 2021), chitosan (Kurek et al., 2018; Li et al., 2021), bacterial cellulose nanofiber (Moradi et al., 2019), and agar/potato starch (Choi et al., 2017). Sodium alginate is a naturally derived hetero-polysaccharide mainly obtained from brown seaweed and consists of varying compositions and sequences of β-D-mannuronic acid (M) and α-L-guluronic (G) with 1,4-glycosidic bonds (Sharma et al., 2022). This polymer exhibits several desirable properties, including low cost, biocompatibility, biodegradability, and ability to form a 3D-network gel referred to as an egg-box structure in the presence of polyvalent or divalent cations (e.g., Ca^2+^) (Arriola et al., 2016).

Even though solvent casting and coating a filter paper or polymeric films with a dye solution are frequently used in preparing pH-indicator films, pH-indicators produced with these techniques must be sufficiently sensitive and fast-responding in most cases. Encapsulating the anthocyanins in alginate beads with electrostatic extrusion provides a high level of versatility in terms of particle size and morphology, depending on operating parameters (Mohammadalinejhad et al., 2023). Alginate beads loaded with anthocyanins offer a high surface-to-volume ratio and a porous network. This results in increased active sites interacting with target components, improved sensitivity, and faster response time. Therefore, anthocyanin-loaded Ca-alginate beads can be used as a loading substrate in colorimetric indicators for intelligent food packaging purposes.

Ammonia (NH_3_), dimethylamine (DMA, (CH_3_)_2_N), and trimethylamine (TMA, (CH_3_)_3_N) are the significant components produced during muscle food spoilage because of microbial activities. These volatile amines are also referred to as total volatile basic nitrogen (TVBN), which is used to measure the freshness of the seafood (Mohammadalinejhad et al., 2020). The most used methods to determine freshness in seafood includes amine extraction, steam distillation, and titration to determine TVBN. Moreover, analysis of spoilage bacteria (*Photobacterium*, *Shewanella*, *Brochothrix* spp, and *Pseudomonas*), ATP degradation products, and detection of volatiles in the headspace with GC-MS are routinely performed. Despite the robustness of these techniques, they are time-consuming, destructive, costly, and require specific equipment and specialists. Therefore, using colorimetric indicators to evaluate seafood freshness and facilitate the decision-making process may be promising (Luo et al., 2021).

Despite the development of colorimetric pH indicator films incorporating anthocyanins, this is the first study to examine the feasibility of integrating alginate hydrogel beads containing anthocyanins from purple corn as a novel indicator into packaging. The present study aimed to explore the potential of purple corn anthocyanins as a material for intelligent packaging. To this end, anthocyanins were encapsulated via electrostatic extrusion into the alginate matrix. Two different formulations of anthocyanin-loaded alginate hydrogel beads were produced, and the color stability and the content of the predominant anthocyanin were evaluated under various temperatures, relative humidities, and light conditions during storage. Furthermore, unlike the existing studies that primarily focused on the sensitivity to ammonia, the limit of detection (LOD) for TMA and DMA was investigated in the current research, thereby contributing to more accurate assessment of freshness in muscle food products.

## 2 Material and methods

### 2.1 Chemicals

Sodium alginate (from *Laminaria hyperborea*, M_w_= 2.74×10^5^ g mol^−1^, Protanal LF 200S) was obtained from DuPont Nutrition and Health (Sandvika, Norway). Cyanidin-3-glucoside (C3G) was purchased from PhytoLab GmbH & Co KG (Vestenbergsgreuth, Germany). Other chemicals were provided by Sigma Aldrich.

### 2.2 ^1^H NMR spectroscopy

The chemical composition of the alginate was analyzed using NMR spectroscopy, according to the standard test method established by the American Society for Testing and Materials (ASTM International, W. C., PA., 2012) and previously described by Grasdalen (1983) and Grasdalen et al. (1979). A mild acid hydrolysis process was used to partially degrade the alginate, achieving an approximate degree of polymerization of 50. The process began by dissolving 15 mg of alginate in deionized water. The pH of the resulting solution was adjusted to 5.6, and then hydrolyzed for 1 h at 95 °C. The solution was cooled to room temperature (RT), and the pH was adjusted to 3.8 followed by hydrolysis for 50 min at 95 °C. In the next step, the solution was cooled, neutralized (pH=6.8–7.5) and freeze-dried. 10 mg of the residual sample was dissolved overnight in 600 μL of D_2_O, along with the addition of 20 μL of 0.3 M triethylenetetramine-hexaacetic acid as a chelating agent. The sample was centrifuged, and the supernatant was transferred to NMR tubes. 3-(Trimethylsilyl)-propionic-acid sodium salt in D_2_O (1%, 5 μL) was added for internal chemical shift reference. ^1^H NMR spectrum was obtained by BRUKER NEO 600 MHz equipped with 5 mm iProbe TBO (Bruker BioSpin AG, Fälladen, Switzerland) and recorded at 83 °C. The spectra were recorded using TopSpin 4.0.7 software (Bruker BioSpin) and processed and analyzed with TopSpin 4.0.7 software (Bruker BioSpin). The chemical composition of alginate and NMR spectrum is shown in Table 1 and Supplementary Fig. 1, respectively.

**Table 1:** Chemical composition of alginate expressed as fractions of β-1,4-D-mannuronic acid (F_M_) and α-1,4-L-glucuronic acid (F_G_), duplets (F_GG_, F_MM_, F_MG/GM_), and triplets (F_MGM_, F_GGG_, and F_MGG/GGM_).

Alginate	F_G_	F_M_	F_GG_	F_MG_=F_GM_	F_MM_	F_GGG_	F_MGG_=F_GGM_	F_MGM_
Protanal® LF 200 FTS	0.65	0.35	0.52	0.12	0.23	0.47	0.05	0.08

### 2.3 Extraction of anthocyanins from purple corn powder

Purple corn powder was purchased from Dragon superfoods, Bulgaria. Extraction of anthocyanins was performed according to Forghani et al. (2022) with slight modifications. Purple corn powder of specific amounts (20%, 26% (%w/w)) was added to deionized water and stirred continuously at 1000 rpm for 1 h at RT. The solution was filtered with Whatman No.1 filter paper followed by centrifugation at 3438×*g* at 4 °C for 10 min. The final aqueous extract was obtained by filtration using a syringe filter (Polyethersulfone, 30 mm, 0.45 μm).

### 2.4 Purple corn extract characterization

#### 2.4.1 Quantitative determination of predominant anthocyanin

Cyanidin-3-glucoside has been identified as the predominant anthocyanin in PCE (de Pascual-Teresa et al., 2002; Yang and Zhai, 2010). PCE was analyzed using an Agilent 2190 HPLC system (Agilent Technologies, USA) equipped with an infinity diode array detector and ACE® 5 C18 column (250 mm × 4.6 mm, pore size 5 μm) with detection at 520 nm. Mobile phases consisted of A, 0.1% (v/v) trifluoracetic acid (TFA) in water, and B, 100% HPLC-grade acetonitrile. The separation was carried out at 25 °C under isocratic condition beginning with 10% B over the initial 5 min. From minutes 5 to 25, the isocratic condition was adjusted to 15% B. Over the next 5 min, the mobile phase composition increased to 18% B, and from minute 30 to 50, it was maintained at 35% B. A post-time of 10 min was allowed before the next injection. The flow rate set to 0.5 mL/min, and the injection volume was 10 μL. The chromatograms obtained were analyzed using cyanidin-3-glucoside as a standard.

#### 2.4.2 UV–Vis spectra and color properties of the extract

The pH sensitivity of PCE was investigated by mixing 700 μL PCE in 5 mL of 10 mM tris buffer solutions (pH 2–12). After 20 min, the UV–Vis spectra (450–700 nm) of the obtained solutions were recorded by a UV–Vis spectrophotometer (UV-1800, Shimadzu, Kyoto, Japan).

Color parameters were determined via a DigiEye Imaging system (VeriVide Ltd, UK), consisting of a cabinet adjusted to match the D65 illuminant and a digital camera (Nikon D7000, 35 mm lens, Nikon Corp., Japan), centrally mounted on top of it. A Digi Eye Digitizer Chart was used to calibrate the system and relate the camera's RGB signals to the CIE specifications. Images were analyzed with DigiPix Software Version 2.9. Total color difference (ΔE) was calculated using the following formula as described by CIE International Commission on Illumination (1994):

(Eq. 1) $$\Delta E=\sqrt{{\left(L_{0}^{*}-L^{*}\right)}^{2}+{\left(a_{0}^{*}-a^{*}\right)}^{2}+({\left(b_{0}^{*}-b^{*}\right)}^{2}}$$

where *L*_0_*, a_0_*, and *b*_0_* are lightness, red–green, and yellow-blue color attributes of diluted PCE, while L*, a*, and b* are the color parameters after buffer addition.

To ensure an accurate comparison and evaluate the influence of pH and dilution on the color parameters of the extracts, control extracts were prepared by diluting with a buffer having the same initial pH as the extracts (5.2 for 26% PCE and 5.6 for 20% PCE). To this end, 5 mL of tris buffer solution with pH values of 5.2 and 5.6 was added to 700 μL of 26% and 20% extracts, respectively. Therefore, *L*_0_*, a_0_*, and *b*_0_* are the color parameters of diluted extracts.

### 2.5 Hydrogel beads production

Anthocyanins were encapsulated within alginate beads using a custom-made electrostatic droplet generator (NTNU, Trondheim, Norway), based on the optimal conditions reported in our previous study (Mohammadalinejhad et al., 2023). Sodium alginate (1% (w/v)) was dissolved in the extracts prepared in section 2.3 and stirred overnight at RT. Five mL of each prepared solution was then extruded into cross-linking bath through a 0.35 mm nozzle (Staedtler Mars GmbH & Co. KG, Nuremberg, Germany) using syringe pump (Graseby Medical Ltd., Watford, Hertfordshire, UK) at a constant flow rate of 20 mL/h. Cross-linking solution consisted of 50 mM CaCl_2_ and 0.1 M HCl in the extract (pH= 2.5). The operating voltage was 5 kV for the 20% extract and 3 kV for the 26% extract. The distance from the nozzle tip to the cross-linking solution was set to 5 cm. After droplet penetration, the beads were left to harden for 30 min to ensure thorough gelation. The beads were then filtered and rinsed with deionized water and 70% ethanol to remove impurities. Table 2 summarizes the conditions in which beads were prepared.

**Table 2:** Conditions in which two different beads (F_1_ and F_2_) were produced.

Sample	Alginate concentration (w/v%)	Extract concentration (w/w%)	Voltage (kV)	CaCL_2_ concentration
F_1_	1%	20%	5 kV	50 mM
F_2_	1%	26%	3 kV	50 mM

### 2.6 Characterization of beads

#### 2.6.1 Moisture content and water activity

The moisture content of the beads was determined gravimetrically by evenly spreading 1 g of the beads in petri dishes. The samples were dried at 70 °C to a constant mass (Horwitz et al., 1970). The beads’ water activity (a_w_) was analyzed using Novasina a_w_ meter (AG, CH-8853 Lachen, Switzerland). The measurements were performed in triplicate.

#### 2.6.2 Particle size

The size of the hydrogel beads was determined through an optical light microscope (Nikon Eclipse TS100). Images of the particles were captured, and the diameter of 100 beads was measured.

#### 2.6.3 Encapsulation efficiency measurement

Encapsulation efficiency (EE) was quantified using the method described by Mohammadalinejhad et al. (2023). For this purpose, 0.125 g wet beads were dispersed in a 4.75 mL solution of 0.2 M EDTA and thoroughly mixed for 30 min at RT to ensure complete dissolution of the beads. Subsequently, the solution was centrifuged at 3438×*g* for 20 min at 4 °C, and the obtained supernatant was filtered using a 25 mm, 0.45 μm syringe filter made of Polyethersulfone (Sigma Aldrich). The C3G content was determined using the HPLC method outlined in section 2.4.1. The EE was calculated by comparing the C3G content of the EDTA solution containing dissolved beads with that of the alginate solution containing PCE (0.125 g) diluted with EDTA.

### 2.7 Storage stability studies

#### 2.7.1 Light stability

The color stability and degradation kinetic of C3G in hydrogel beads was evaluated under light (11700 ±100 Lux) at ambient (23 °C) and chilled storage temperatures of commercial food products (4 °C). Random samples were collected at specific time intervals (0, 1,3, 5, 7, 14, and 21) and color parameters (CIE Lab) were determined with DigiEye imaging system as described in section 2.4.2. The degradation kinetic of C3G during storage followed a first-order reaction (R^2^> 0.9) (Mohammadalinejhad et al., 2023). A plot of C3G retention (%) versus time (day) was used to calculate the kinetic rate constant (k) at various temperatures according to Eq. (2):

(Eq. 2) $$C=C_{0}\;\exp\left(-kt\right)$$

where C is the concentration of cyanidin-3-glucoside (mg C3G/g wet bead) at time t, and C_0_ represents the initial concentration of C3G. The half-life (t _½_) of C3G during storage was calculated as follows:

(Eq.3) t12=Ln(0.5)k

#### 2.7.2 Stability under different relative humidity levels

The stability test under different relative humidity (RH) levels was assessed at RT and 4 °C. Desiccators with two relative humidity levels, 53%, and 97% were obtained by saturated magnesium nitrate and potassium sulfate solutions, respectively. RH was monitored and verified in each chamber at equilibration with a hygrometer (Beurer HM16 Hygrometer, Switzerland). Hydrogel beads were placed in each desiccator and kept in the dark. C3G content, a_w,_ and color properties were determined on days 0, 1,3,5, 7, 14, and 21. The experiment was carried out in triplicate.

### 2.8 Sensitivity to volatiles

Hydrogel beads were investigated for their ability to discriminate three volatile amines. To assess the color response of the fabricated indicators to ammonia, TMA, and DMA, beads were placed on a permeable fabric and attached to the mouth of Scotch bottle containing 25 mL various concentrations of ammonia (1, 3, 6, 12, 21, 30, 45, 60, 120, and 300 ppm), DMA and TMA (5, 10, 20, 40, 75, 150, 250, 500, 1000, and 2000 ppm). The exposure was done for 24 h, and colorimetric values were recorded. The limit of detection (LOD) and limit of quantification (LOQ) were calculated based on the total color difference versus volatile concentration (ppm) in the linear range from the formulas below (Luo and Lim, 2020):

(Eq.4) LOD= 3σ/S

(Eq. 5) LOQ= 10 σ/S

Where, σ and S represent the root-mean-square deviation of blank measurements and the slope of the fitted line of the calibration curve, respectively.

### 2.9 Statistical analysis

Each experiment was performed in triplicate, and the results were reported as the mean value with the standard deviation (SD). In the stability studies, temperature and storage time were considered the primary factors and analyzed through the general linear model (GLM). One-way analysis of variance (ANOVA) was carried out to evaluate the sensitivity to volatiles and colorimetric change at various pH levels. The difference was considered statistically significant if p < 0.05, and the post hoc Tukey's test was conducted using SPSS software (version 28.0.1.0 (142)). Two anthocyanins-loaded hydrogels were compared by independent sample *t*-test.

## 3 Results and discussion

### 3.1 UV-VIS spectra and color properties of the extract

**Fig. 1:**
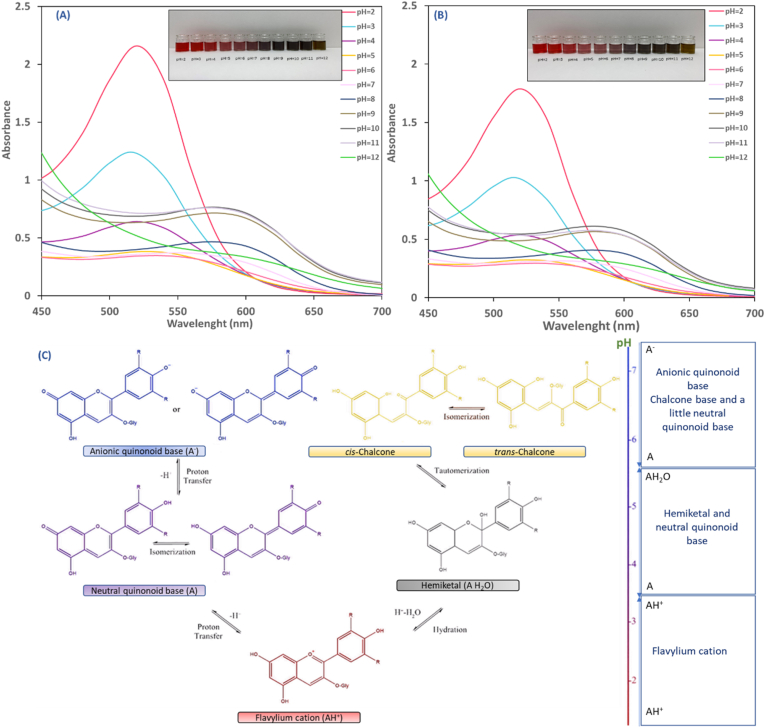
Color change and UV–Vis spectra of aqueous extracts of 26% (A) and 20% (B) purple corn powder at different pH levels ranging from 2 to 12; Structural transformation of anthocyanins affected by the various pH levels (C (Chen et al., 2022),). (For interpretation of the references to color in this figure legend, the reader is referred to the Web version of this article.)

The concentration of C3G was found to be 5.28±0.1 ^mg^/_100 mL_ and 6.33±0.1 ^mg^/_100 mL_ in 20% and 26% extract, respectively. The color change of PCE was examined under various buffer solutions to confirm its suitability as a pH indicator. Fig. 1 (A-B) depicts the changes in visual color and spectral properties of the PCEs (20% and 26%) in response to varying pH levels. The PCEs were bright red at pH 2–3, while at pH 4–6, they appeared pink, and at pH 7, they turned purple. As the pH was raised to 8, the color changed to greyish purple, and the grey color intensified up to pH 11. At pH 12, the color of the solution further changed to a seaweed green. Various studies have reported a wide range of colors in anthocyanin extracts under different pH conditions. These color variations can be attributed to factors such as the concentration of anthocyanins, solubility, stability, presence of a mixture of anthocyanidins, and interaction of anthocyanins with other components (Ghorbani et al., 2021; He et al., 2022). Generally, color changes occur because of structural modifications of the anthocyanin molecules induced by variations in pH levels. These structural changes include the formation of the flavylium cation (at pH below 3), which is relatively stable due to the widely delocalized positive charge marked at O_1_. Under mild acidic conditions, flavylium cation undergoes a competitive process, including hydration and deprotonation, leading to hemiketal and neutral quinonoid base formation as depicted in Fig. 1 (C) (Chen et al., 2022). The hemiketal is in a fast cycle-chain equilibrium with two tautomers, namely, cis-chalcone (C_cis_) and trans-chalcone (C_trans_), which exhibit a yellow color. The neutral quinonoid bases, on the other hand, transform into anionic quinonoid bases as the pH increases towards neutrality via losing a second proton (Chen et al., 2022; He et al., 2022).

The pH of the anthocyanin solution affects the wavelength of the maximum absorption peak and intensity (Alizadeh-Sani et al., 2021). Absorption peaks were observed between 516 and 522 nm within the pH range of 2–3, with 26% PCE exhibiting higher absorbance than 20% PCE. As the pH increased, the height of these peaks decreased. Likewise, Dikmetas et al. (2023) and Zeng et al. (2023) observed a moderately higher absorption in red cabbage and black rice extract under acidic conditions, respectively. Increasing the alkalinity from 4 to 8 resulted in a bathochromic shift along with decreased absorbance in the absorption maxima of PCEs, with the peak moving from 522 in 26% PCE and 520 in 20% PCE to 574 nm. An increase in pH from 8 to 11 led to a gradual rise in absorbance. Similar UV–Vis observations were reported by He et al. (2022) and Luchese et al. (2018) using purple sweet potato and blueberry residue anthocyanins, respectively.

**Fig. 2:**
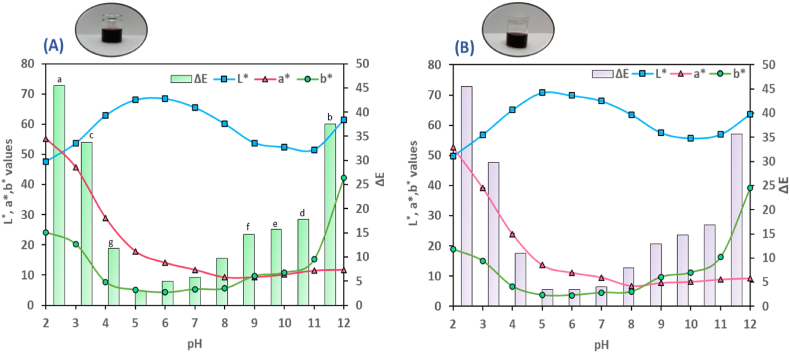
Aqueous extracts of 26% (A) and 20% (B) purple corn powder along with their color properties in response to various pH ranges (2–12). Different letters show a significant difference (p<0.05, One-way ANOVA, followed by Tukey's post hoc test). (For interpretation of the references to color in this figure legend, the reader is referred to the Web version of this article.)

CIE Lab color parameters of freshly prepared PCE at 20% and 26% were compared, and results indicated a significant difference (p< 0.05) in color values between the two concentrations. As expected, the 20% PCE exhibited lighter color (L*= 67.48±0.08) with lower redness (a*=13.58±1.18) and yellowness (b*=4.15±0.02) values than the 26% PCE (L*= 65.12±0.1, a*=17.56±0.02, b*=5.39±0.02). The color properties (L*, a*, b*, and ΔE) of the two extracts at pH solutions ranging from 2 to 12 are depicted in Fig. 2. The lightness (L*) values of the extracts showed a significant increase, reaching a maximum of 68.08 in the 26% PCE (Figs.. 2A) and 70.78 in the 20% PCE (Fig. 2B) at pH 5, followed by a decrease until pH 11. However, at pH 12, the values increased (p< 0.05) again in both extracts. The redness (a*) significantly decreased (p< 0.05) with an increase in pH and reached its minimum level of 9.29 in the 26% extract and 6.77 in the 20% extract at pH 8. The yellowness (b*) of the extracts exhibited a notable decrease from pH 2 to 6, followed by a gradual increase from 6 to 11, and eventually, a significant surge that peaked at pH 12, with the 26% PCE reaching a maximum value of 42.26 and the 20% PCE at 39.33. The colorimetric analyses aligned with visual color changes in Fig. 1 (A-B), and the trends observed were consistent with the findings of previous studies (do Carmo Brito et al., 2017; Moradi et al., 2019). The total color difference (ΔE) between the fresh PCEs and the PCEs at various pH levels is illustrated in Fig. 2A-B. The pH of the extracts after extraction was 5.2± 0.2 in 26% PCE and 5.6±0.1 in 20% PCE, which explains why the extracts had the lowest ΔE values of 2.95 and 3.44 at pH 5 for 26% and 20% PCE, respectively. However, at lower and higher pH levels, the ΔE values were significantly higher than those at pH 5 p> 0.05). A human eye can easily detect a ΔE value exceeding 5, while values above 12 can be noticed by untrained individuals (Prietto et al., 2017). These results suggest that purple corn anthocyanins could potentially serve as an indicator in intelligent packaging, as the extract demonstrated a clear colorimetric response to both acidic (pH<4) and alkaline environments (pH>7).

### 3.2 Anthocyanins-loaded hydrogel beads characterization

The moisture content of F_1_ and F_2_ hydrogel beads was over 96% and 93%, respectively, with the latter showing higher solids content (Table 3). Current observations align with those of Nguyen et al. (2022), who noted a negative relationship between moisture content and anthocyanin levels in alginate beads containing roselle anthocyanins. The solids content of F_2_ alginate hydrogel beads was significantly higher compared to F_1_. This difference can be attributed to the superior adsorption capacity of the porous matrix within F_2_ beads and the utilization of a higher concentration of the extract during their preparation. Previous findings from our study (Mohammadalinejhad et al., 2023) revealed that F_2_ possess larger pore size and volume, which might facilitate the rapid filling of empty spaces within the hydrogel bead structure through efficient adsorption of the purple corn extract. Consequently, a higher quantity of solid components was introduced to the samples. These observations are further supported by the encapsulation efficiency results (Table 3). The size of F_1_ hydrogel beads was 1142 μm, which was significantly smaller than the particle size of F_2_ beads, which were found to be 1912 μm. The voltage used during the electrostatic extrusion of these beads was identified as the most significant factor influencing their particle size (Mohammadalinejhad et al., 2023). Changing the voltage during the production process resulted in different sizes of alginate beads. Increasing the voltage led to the formation of smaller beads, whereas decreasing the voltage yielded larger beads (Mohammadalinejhad et al., 2023). This flexibility in voltage allowed for the achievement of the desired particle size while maximizing encapsulation efficiency. It was postulated that the two types of beads would exhibit varying storage stability and sensitivity to volatile amines in practical application due to their different physiochemical properties.

**Table 3:** Characterization of anthocyanins-loaded hydrogel beads in terms of total solids content, water activity, Cyanidin-3-glucoside (C3G) content, and particle size.

Sample	Total solids content (%)	C3G content (mg/g wet bead)	Encapsulation efficiency (%)	Water activity	Particle size (μm)
F_1_: (1% Alg-20% PCE-5 kV)	3.9±0.5^b^	0.045±0.000^a^	70.2±5.9^b^	0.938±0.002^a^	1142±142^b^
F_2_: (1% Alg-26% PCE-3 kV)	6.5±0.5^a^	0.055±0.001^b^	81.7±5.7^a^	0.949±0.009^a^	1912±32^a^

Different superscript letters within each parameter and column indicate significant differences (p< 0.05), evaluated by One-Way ANOVA and Tukey post hoc test.

### 3.3 Stability studies

#### 3.3.1 Cyanidin-3-glucoside retention: effect of time, temperature, and light

**Fig. 3:**
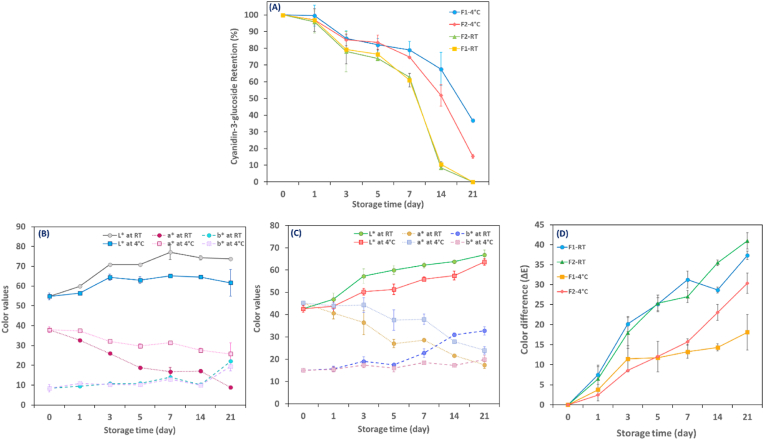
Cyanidin-3-glucoside retention of anthocyanins-loaded hydrogel beads (A); L*, a*, and b* color values of F_1_ indicator(B), F_2_ indicator (C); ΔE values of fabricated indicators (D) indicators under light during storage at RT and 4 °C. Abbreviations: F_1_: 1% Alginate-20% extract-5 kV and F_2_: 1% Alginate-26% extract-3 kV. (For interpretation of the references to color in this figure legend, the reader is referred to the Web version of this article.)

Indicators F_1_ and F_2_, containing initial cyanidin-3-glucoside contents of 0.045 ^mg^/_g wet bead_ and 0.055 ^mg^/_g wet bead_, respectively, were stored at both refrigerated and room temperatures for 21 days under light. The results revealed a significant impact of storage time and temperature on the C3G retention (p < 0.05). The retention of C3G in both hydrogels decreased to approximately 10% of the initial concentration at RT on day 14. It continued to fall until nearly zero at the end of the 21 days (Fig. 3A). However, F_1_ and F_2_, in turn, retained 67% and 52% of the initial C3G concentration at 4 °C after 2 weeks of storage. The precise mechanism of thermal degradation is not yet fully understood. Nevertheless, it is believed to involve the formation of chalcones as an initial step, as well as the loss of glycosyl moieties and the formation of α-diketones. As the degradation progresses, various end products form, such as benzoic acid derivatives, coumarin derivatives, and trihydrobenzaldehyde. These end products are generated through a series of chemical reactions that involve the breakdown of anthocyanin molecules and the rearrangement of their chemical structure (Reyes and Cisneros-Zevallos, 2007).

Although no significant difference was found in the C3G retention of F_1_ and F_2_ at 4 °C until day 14, a noticeable disparity was recorded by the end of storage, with 37% and 15% retention rates observed for F_1_ and F_2_, respectively (Fig. 3A). The differences in retention could be due to variations in the morphological structure, pore size, and pore volume of alginate beads (Mohammadalinejhad et al., 2023).

Table 4 presents the degradation rate constant (k) and half-life value (T_1/2_) of C3G in F_1_ and F_2_ subjected to light and under dark conditions. The results displayed that lowering the storage temperature led to a significant increase in the half-life of the C3G, as well as a reduction in the degradation rate constant (k) (p< 0.05). Furthermore, it was observed that the light-exposed samples were less stable compared to samples stored in the dark. The half-life of C3G in F_1_ hydrogel exposed to light at refrigerated conditions was shorter, with a value of 15.9 days, compared to the same samples stored in the dark, where the half-life was estimated to be approximately 10 times longer, 157 days. Similarly, for F_2_, the half-life was 8.43 days when exposed to light and 81.8 days when stored in the dark. The photochemical degradation mechanism of anthocyanins is a complex process that involves various reactions and intermediates. When anthocyanins are exposed to light, they absorb energy and are excited to a higher energy state. This excited state can lead to several different reactions that result in the degradation of the molecule. One proposed mechanism involves the formation of a C_4_ adduct intermediate, which is formed by the hydrolysis of position C_4_ in the flavylium excited state. This intermediate can then undergo subsequent hydrolysis and ring opening to create various degradation products, including aromatic aldehydes, carboxylic acids, and phenolic compounds. Another suggested mechanism involves the hemolytic scission of the C_2_–C_3_ bond of the chalcone form, which can lead to the formation of various degradation products. This reaction is postulated to occur via a radical pathway, which involves the formation of a radical intermediate that can undergo multiple subsequent reactions (Furtado et al., 1993). Maccarone et al. (1987) explained the photochemical degradation of anthocyanins from flavylium cation via carbinol pseudo-base to chalcone. It is not feasible to verify the exact mechanism of photodegradation in alginate hydrogel beads containing anthocyanins due to the large variety of the compounds in the extract along with the lack of information on equilibrium constants for acylated forms. Hence, the exact mechanisms involved in the photochemical degradation of anthocyanins may vary depending on the anthocyanin molecule, temperature, and light intensity.

**Table 4:** The degradation rate constant (*k, 10*^*−2*^*/day*) and half-Life (*T*_*1/2*_*, day*) of hydrogel beads (F_1_ and F_2_) containing anthocyanins stored at room temperature (RT) and 4 °C under light and dark conditions.

Parameter	Samples exposed to light				Samples stored in dark conditions (Mohammadalinejhad et al., 2023)			
	F_1_		F_2_		F_1_		F_2_	
Temperature	RT	4 °C	RT	4 °C	RT	4 °C	RT	4 °C
k (10^−2^/day)	21.8	4.3	22.2	8.2	12.3	0.45	1.89	0.94
T_1/2_ (day)	3.17	15.9	3.11	8.43	56.4	157.0	36.6	81.8
R^2^	0.94	0.91	0.94	0.9	0.94	0.87	0.86	0.92

Abbreviations: F_1_: 1% Alginate-20% extract-5 kV and F_2_: 1% Alginate-26% extract-3 kV.

#### 3.3.2 Color stability under light as a function of time and temperature

Color stability is of utmost importance in pH-sensitive indicators as it ensures that the indicator retains its color throughout the entire shelf-life of the product and provides consumers with accurate and reliable visual feedback (Mohammadalinejhad et al., 2020). The effect of storage temperature (RT and 4 °C) on the color stability of anthocyanin-loaded hydrogel beads was investigated under an accelerated light intensity of 11700 ± 100 lux over a 21-day period. The results revealed a significant impact of temperature (p< 0.05) on the maintenance of the beads' original color, with the samples showing more stable color when stored at 4 °C.

The color parameters L*, a*, and b* were measured to evaluate various aspects of color, including lightness (L* 100/0 white/black), redness (+a*), greenness (-a*), yellowness (+b*), and blueness (-b*). On day 0 of storage, the hydrogel beads exhibited L*, a*, and b* values of 54.73 ± 1.71, 37.88 ± 1.47, and 8.39 ± 1.84 for F_1_ (Figs.. 3B) and 42.45 ± 1.49, 45.20 ± 0.60, and 15.03 ± 0.14 for F_2_ (Fig. 3C). Based on the figures, the brightness (L*) of both indicators increased (p< 0.05) during storage, although the effect was more pronounced at RT compared to 4 °C. The a* values for F_1_ and F_2_ decreased by 76% and 61%, respectively, after 21 days of storage at RT, while under dark storage conditions, the reductions were reported to be only 23% and 19% for F_1_ and F_2_, respectively, after 4 weeks according to our earlier study (Mohammadalinejhad et al., 2023). At 4 °C, the decline in a* value was slower, with a reduction of 32% for F_1_ and 47% for F_2_. These findings align with previous studies by Abdin et al. (2021) and Fan et al. (2019), which reported a decrease in redness due to anthocyanin degradation. Both hydrogel beads containing anthocyanins exhibited a notable increase (p< 0.05) in the b* value at all temperatures as the duration of storage increased. This rise indicated a higher degree of yellowness, likely due to the formation of chalcone species (Ratanapoompinyo et al., 2017). The ΔE values exhibited an upward trend as the storage duration extended, indicating a decline in color stability over time (Fig. 3D). The ΔE values of F_2_ displayed an increasing trend, especially after one week at 4°. In contrast, the ΔE values of F_1_ showed a continuous increase until day 3, followed by a relatively stable trend thereafter. These findings suggest that the smaller hydrogel beads (F_1_) are less susceptible to noticeable color changes compared to F_2_ as time extended. Therefore, it is recommended to apply the F_1_ freshness indicator for muscle food products with both short and long shelf lives. On the other hand, the F_2_ indicator is most suitable for highly perishable muscle foods.

When comparing the ΔE values of anthocyanin-loaded alginate hydrogel beads stored at 4 °C for 21 days under light with those in our previous study (Mohammadalinejhad et al., 2023) with the same formulation but kept in the dark, a notable difference in color retention can be observed. Indeed, light exposure accelerated the deterioration of color in F_1_ by 3.7 times and in F_2_ by approximately 6 times. The degree of anthocyanin acylation and the storage conditions affect the color stability of pH indicators (Alamdari et al., 2022). Highly acylated cyanidin-based anthocyanins, such as those found in red cabbage (Prietto et al., 2017) and black carrots (Moradi et al., 2019), typically exhibit excellent color stability. Purple corn powder appears to have low acyl groups and, therefore, indicated moderate color stability. The medium to low stability could also be related to the high moisture content of hydrogels leading to the hydration of anthocyanins.

#### 3.3.3 Color stability under different relative humidity levels

Bacterial metabolism and growth can influence the humidity level inside packaged food. Hence, an indicator that maintains a stable color at any humidity level is crucial to ensure accurate detection (Li et al., 2022a).

**Fig. 4:**
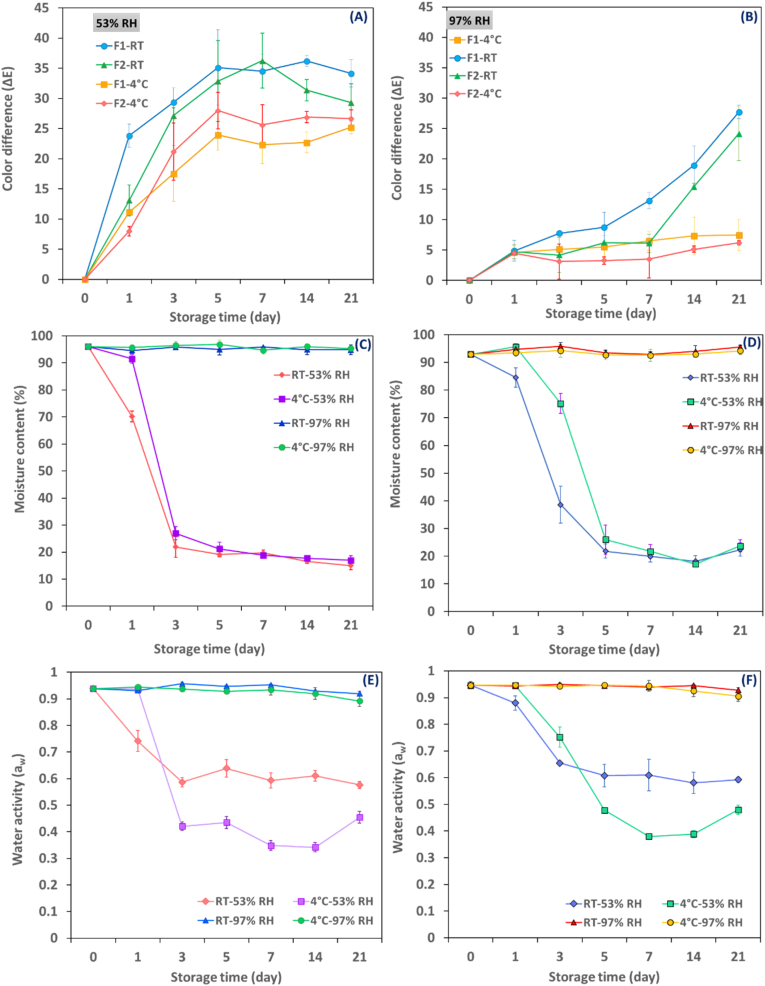
ΔE values of anthocyanins-loaded hydrogel beads during storage at RT and 4 °C at 53% RH (A); 97% RH (B); Moisture content and water activity of F_1_ (C and E) and F_2_ (D and F) hydrogel beads at different relative humidity levels and temperatures during 3 weeks of storage. Abbreviations: F_1_:1% Alginate-20% extract-5 kV and F_2_: 1% Alginate-26% extract-3 kV.

Fig. 4(A-B) depicts the color stability of the hydrogel beads at relative humidity levels of 53% and 97% under ambient and refrigerated temperatures. The results indicate that as the temperature increased, the hydrogel beads showed a significant increase in ΔE values at both RH levels over the storage period (p<0.05). The hydrogel beads stored at 53% RH exhibited a significantly higher color difference than those held at 97% RH at all temperatures (p< 0.05). The mean ΔE value of the samples stored at 53% RH ranged between 8 and 23 after one day and increased to 25–34 by the end of the storage period, depending on the type of hydrogel and temperature (Fig. 4A). On day 5, at RT, the hydrogel beads stored at 53% RH showed the highest color difference, with F_1_ and F_2_ revealing values of 35 and 33, respectively. In contrast, at 4 °C, F_1_ and F_2_, in turn, had mean ΔE values of 24 and 28, and lower changes were observed after that (Fig. 4A). When the hydrogel beads with high moisture content were placed at 53% RH at RT, F_1_ and F_2_ lost 85% and 77% of their initial moisture content, respectively, and 83% and 76% at 4 °C by the end of the 21-day storage period (Fig. 4C-D). Moisture loss of alginate hydrogel beads under low relative humidity has also been reported in other studies (Łabowska et al., 2023; Tay et al., 2017). The significant mass loss caused the hydrogel beads to shrink and change shape, resulting in very dark beads unsuitable for intelligent packaging of food products with low relative humidities, such as salted and dried fish packaging. On the other hand, both types of hydrogel beads stored at 97% RH and 4 °C showed a steady trend (Fig. 4B) between day 1 and 21 (|ΔE| ≤ 3), indicating high color stability of produced anthocyanins-loaded alginate hydrogel beads. According to these results, both F_1_ and F_2_ could be recommended for use in extended storage periods of muscle food but only in RH< 90% inside the package.

#### 3.3.4 Water activity as a function of relative humidity and temperature

Fig. 4E-F shows the changes in a_w_ for F_1_ (Fig. 4E) and F_2_ (Fig. 4F) exposed to 53% and 97% RH at two different temperatures. A_w_ of both samples was affected by relative humidity and storage time (p> 0.05). However, the temperature significantly impacted the water activity only under lower RH (53%), regardless of the type of beads investigated. A steady trend with negligible fluctuations in a_w_ was observed at a 97% RH in all studied temperatures. Anthocyanins-loaded alginate hydrogel beads stored at 53% RH underwent moisture loss to achieve equilibrium with the surrounding environment. F_1_ showed a rapid reduction in moisture in comparison to F_2,_ which is due to their higher surface-to-volume ratio and smaller particle size (Table 3; Fig C–D). F_1_, with an initial a_w_ of 0.93, reached values of 0.57 and 0.45 on day 21 when stored at RT and 4 °C, respectively. In comparison, F_2_ displayed a slightly lower reduction in a_w_, with values decreasing from 0.94 to 0.59 at RT and 0.48 at 4 °C. In other words, a_w_ in F_2_ was somewhat higher than F_1,_ which can be attributed to higher anthocyanins content that can be bonded with water and impede moisture loss.

According to the results, an increase in a_w_ can be seen with increasing temperature in both samples, which can be due to the greater molecular mobility at higher temperatures. This observation was only found after reaching equilibrium moisture content on day 3 for F_1_ and two days later for F_2_ on day 5 (Fig. 4E-F). It should be noted that before reaching equilibrium, moisture content controlled the water activity in a way that hydrogel beads stored at 4 °C showed more significant water activity than those at RT because of high moisture content (Tonon et al., 2010).

#### 3.3.5 Cyanidin-3-glucoside retention under simulated RH of seafood package

Hydrogel beads containing anthocyanins are a promising material for intelligent food packaging applications, particularly for products with high relative humidity inside the package, such as fresh fruits, vegetables, and meat products (He et al., 2023). Hydrogel beads were placed in desiccators with two different relative humidity levels (53% and 97%) and stored in darkness at RT and 4 °C. The content of C3G content was analyzed by HPLC.

As mentioned earlier, under lower relative humidity (53%), hydrogel beads containing anthocyanins tend to dry out, shrink, and darken, which makes them less suitable for use. Therefore, the focus of the study was to investigate the retention of C3G under simulated condition of 97% relative humidity.

The retention of C3G in both hydrogels was significantly influenced by temperature and storage time, with a notable interaction effect between the two factors (p< 0.05). As the temperature increased, the retention of C3G significantly decreased (p< 0.05), leading to approximately 40% greater C3G loss by the end of day 21 at RT compared to storage at 4 °C for the same samples. Anthocyanin degradation under current storage conditions is likely related to the cleavage of covalent bonds, which form colorless compounds as temperature increases. It can be postulated that the water molecules in the hydrogel structure accelerates the rate of hydrolysis and break down the glycosidic bonds, resulting in the loss of red color (Adams, 1973). Apart from hydrolysis, C3G can undergo polymerization with other anthocyanins or phenolic compounds in the extract. Moreover, the presence of proteins and reducing sugars in the extract can induce the Maillard reaction, commonly occurring at high temperatures during storage. This reaction results in the condensation of anthocyanins with hydroxymethylfurfural, which could cause the formation of brown-colored compounds (Kuck et al., 2017).

**Fig. 5:**
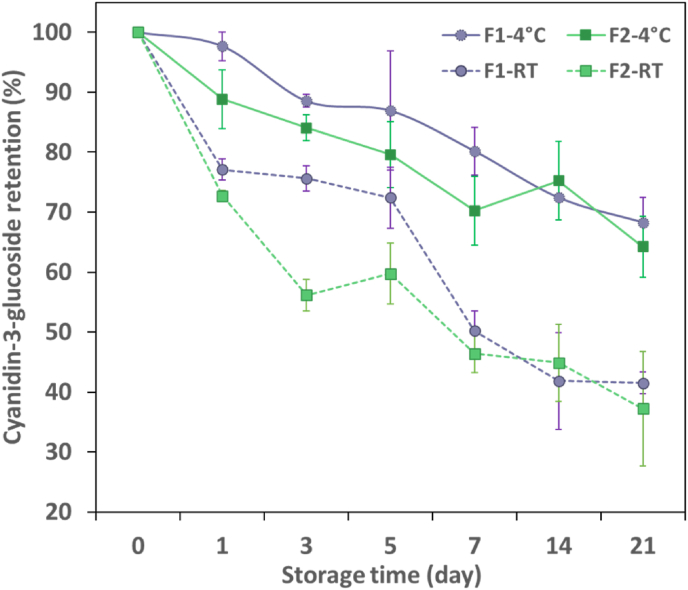
Cyanidin-3-glucoside retention in two different alginate hydrogel beads at RT and 4 °C under 97% relative humidity. Abbreviations: F_1_:1% Alginate-20% extract-5 kV and F_2_: 1% Alginate-26% extract-3 kV.

The concentration of C3G in F_1_ and F_2_ significantly decreased (p< 0.05) over the storage period, with F_1_ showing better retention than F_2_ at both stored temperatures. At refrigerated temperature, the concentration of C3G in F_1_ significantly declined after 7 days, reaching 80% of the initial concentration, while F_2_ showed a significant reduction after 5 days, with 79% of the initial concentration remaining (Fig. 5). Despite these losses, both samples maintained over two-thirds of their initial C3G concentrations by the end of the storage period, with F_1_ and F_2_ showing a concentration of 68% and 64% of initial concentration, respectively. It can be assumed that both F_1_ and F_2_ would contain an adequate amount of C3G within the alginate matrix, ensuring their ability to remain responsive in an intelligent packaging of products with high RH% inside the package during storage at 4 °C in week 3. F_1_ showed statistically significant retention of C3G than F_2_ regardless of temperature. The difference can be associated with the physiochemical properties of these two beads, such as moisture content, a_w_, particle size, pore size, and pore volume. As presented in Fig. 4E-F, both hydrogel beads showed almost the same water activity at 97% RH, characterizing the available water to the chemical reactions during the storage (Syamaladevi et al., 2011). The effect of particle size on anthocyanins retention was investigated by de Moura et al. (2018), who proposed that smaller particles, with their increased surface-to-volume ratio, provide more active sites for anthocyanins-water reaction. However, our results did not align with this hypothesis, as the smaller hydrogel beads (F_1_) provided better protection. Instead, pore volume and pore size appear to play a critical role in retention of C3G as larger pores facilitate water diffusion and promote interactions with anthocyanins. The pore volume and pore size of F_1_ were 0.08 cm^3^/g and 17.4 nm, respectively, while F_2_ had a pore volume of 0.44 and a pore size of 34.68 nm (Mohammadalinejhad et al., 2023). Based on our findings, smaller pores restrict water penetration, leading to slower hydrolysis rates and anthocyanin retention.

### 3.4 Sensitivity to volatiles

The pH of the packaging headspace is influenced by volatile metabolites (NH_3_, DMA, and TMA) produced by microbial activities during the spoilage of protein-rich food products. Thus, the relatively high sensitivity of hydrogel beads to the targeted analytes might reflect the indicator's potential to respond to pH changes.

#### 3.4.1 Ammonia sensitivity

**Fig. 6:**
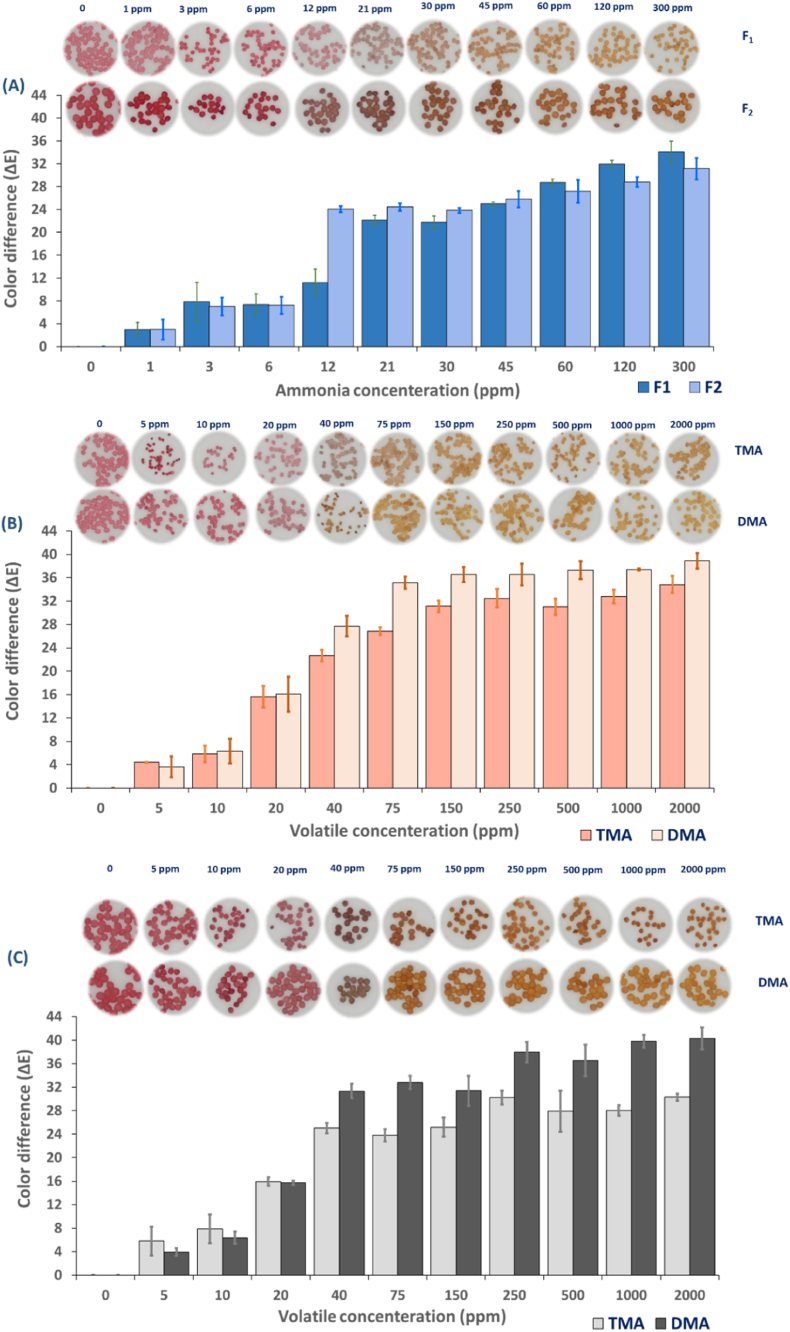
Corresponding ΔE values of fabricated indicators and representative images of them upon exposure to volatile amines; Ammonia (A); The color response of F_1_ to DMA and TMA (B); The color response of F_2_ to DMA and TMA (C). Abbreviations: F_1_: 1% alginate-20% extract-5 kV, F_2_: 1% alginate-26% extract-3 kV. (For interpretation of the references to color in this figure legend, the reader is referred to the Web version of this article.)

**Fig. 7:**
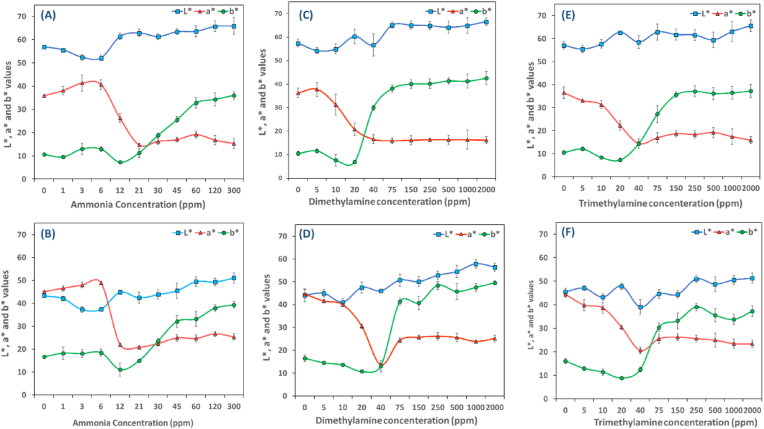
Color parameters of anthocyanin-loaded hydrogel beads after being exposed to Ammonia (A–B), Dimethylamine (C–D), and Trimethylamine (E–F). The upper and lower columns correspond to F_1_ and F_2_, respectively. Abbreviations: F_1_: 1% alginate-20% extract-5 kV, F_2_: 1% alginate-26% extract-3 kV. (For interpretation of the references to color in this figure legend, the reader is referred to the Web version of this article.)

Anthocyanin-loaded alginate hydrogel beads were investigated for their responsiveness to ammonia by placing them on a permeable fabric attached to the mouth of a bottle. The beads were exposed to different concentrations of ammonia ranging from 1 to 300 ppm for 24 h, and color values were recorded. Fig. 6A depicts images of two different alginate hydrogel beads containing anthocyanins after exposure to various concentrations of NH_3_ compared with the image before exposure to NH_3_. All the beads indicated good color uniformity and a significant change from pink to yellow by rising concentration. The color change mechanism of the hydrogel beads is the alkaline environment induced by the hydration and hydrolysis of ammonia vapor (NH_3_ + H_2_O→ NH_4_^+^+ OH^−^). NH_4_^+^ creates an alkaline environment on the surface of the bead. This causes the phenolic hydroxyl group to undergo an acid-base reaction with OH^−^ and, ultimately, the formation of a phenolic oxygen anion in the anthocyanin structure (Ma et al., 2017). Lightness (L*) and yellowness (b*) enhanced (p< 0.05) in both indicators as the ammonia concentration elevated (Fig. 7 (A-B)), while redness (a*) increased only until 6 ppm and faded afterward (p< 0.05). Visual color in sample F_1_ varied from rose pink to pale pink when ammonia concentration was elevated from 0 to 12 ppm (Fig. 6A). Then beige-brown appeared at 21 ppm, and afterward, beads became yellow with increasing NH_3_ concentration. The color in F_2_ was dark pink before exposure to ammonia gas and then turned pinkish brown at 12 ppm ammonia. The color then changed to brown at 30 ppm, followed by the appearance of yellow color, which intensified as the ammonia concentration increased up to 300 ppm. Fig. 6A shows no discernible color change as the concentration increased until 1 ppm in both indicators (ΔE> 4). Color difference (ΔE) in the F_2_ indicator rose steeply from 6 to 12 ppm (234% increase), whereas an increase of 127% was recorded in F_1_ in higher concentrations, between 12 and 21 ppm. ΔE values of F_1_ and F_2_ reached their maximum at 300 ppm, corresponding to 34 and 31, respectively.

The limit of detection (LOD) and limit of quantification (LOQ) refer to the lowest concentration of an analyte that can be calculated reliably through a specific method of measurement (Sukhavattanakul and Manuspiya, 2021). These data are presented in Table 5. A linear relationship between ΔE values and NH_3_ concentrations was observed between 0 and 12 ppm. F_2_ was found to be more sensitive than F_1_ to ammonia. LOD values were reported to be 25 and 15 ppm for F_1_ and F_2,_ respectively. Meanwhile, LOQ values were 83 and 50 ppm. R^2^ values above 0.9 indicate the calibration curve's high detection sensitivity and precision (Teymouri and Shekarchizadeh, 2022). Several factors can impact the responsiveness and sensitivity of anthocyanins-loaded hydrogel beads, including a_w_, particle size, structural properties (morphology, pore volume, and pore size), anthocyanins content, initial color, and degree of lightness. Based on the morphological observation in our earlier study (Mohammadalinejhad et al., 2023), the F_2_ indicator revealed larger cracks on the surface that might ease the vapor diffusion. In addition, higher a_w_ in F_2_ promotes the reaction between H_2_O and NH_3_, producing more OH^−^, leading to stronger alkaline conditions and rapid color change (Liu et al., 2019). Structural properties and a_w_ possibly play critical roles up to a certain point. Once the threshold concentration (∼120 ppm) is reached, lighter-colored hydrogel beads become more effective at detecting color changes. This implies that the smaller the diameter of the beads, the lighter they appear, resulting in a more efficient detection, particularly at higher concentrations.

**Table 5:** Limit of detection (LOD) and limit of quantification (LOQ) of two different beads to ammonia (NH_3_), Trimethylamine (TMA), and Dimethylamine.

Type of volatile	Sample	Equation	LOD (ppm)	LOQ (ppm)	R^2^
**NH**_**3**_	F_1_	y=0.9314x+1.9124	24.85	82.85	0.94
	F_2_	y=1.8714x+0.0175	14.96	49.85	0.94
**TMA**	F_1_	y=0.6048x+1.804	48.41	161.38	0.96
	F_2_	y=0.5708x+1.147	48.02	167.41	0.98
**DMA**	F_1_	y=0.7047x+0.1808	47.65	158.85	0.99
	F_2_	y=0.7905x+0.3939	47.50	158.34	0.99

Abbreviations: F_1_:1% Alginate-20% extract-5 kV; F_2_: 1% Alginate-26% extract-3 kV; TMA: Trimethylamine; DMA: Dimethylamine.

F_1_ and F_2_ indicators, in turn, were at least 8 and 13-times more sensitive to NH_3_ than cellulose fiber embedded with red cabbage anthocyanins (Nafady et al., 2021). Pirayesh et al. (2023) determined the ammonia detection limit with instrumental color analysis and naked-eye inspection. The values were 50 and 150 ppm, 50 and 150 ppm, and 25 and 50 ppm for nanocellulose hydrogels integrated with Liriope, Aronia, and red cabbage anthocyanins, respectively. In another study (Li et al., 2022b), Polylactic acid (PLA) electrospun nanofiber incorporated by blueberry anthocyanins showed an LOD of 35.39 ppm after 5 min exposure.

According to our findings, it can be concluded that F_2_ is more sensitive to lower concentrations of ammonia, making it an ideal indicator in the early stages of spoilage or in products that lower amount of ammonia is produced. However, indicator F_1_ is preferred in the later stages of the product shelf-life or in products with high level of ammonia production during storage owing to the higher ΔE values and apparent color changes at higher concentrations.

#### 3.4.2 TMA and DMA sensitivity

TMA and DMA are responsible for the fishy odor in seafood products. They are generated from the reduction of TMAO by bacteria such as *Shewanella and Photobacterium* ssp, capable of anaerobic respiration (Hoel et al., 2022). The type of seafood, storage conditions, seasonal variations, and location significantly affect the amount and type of amine compounds produced during spoilage. This is because of the various microorganisms or endogenous precursors involved. Hence, a colorimetric indicator must be tailored for a specific type of seafood product (Luo et al., 2022b).

The color response of hydrogel beads to TMA and DMA was evaluated, and the results are presented in Fig. 6 (B–C). When exposed to the gases, the color of hydrogel beads transitioned from pink to yellow as amine concentration increased from 0 to 2000 ppm. An increase in TMA and DMA concentration significantly influenced the CIE Lab parameters (p< 0.05), resulting in an increase in the brightness (L* value). Conversely, a decrease in the redness (a* value) was observed, while the yellowness (b* value) was enhanced (Fig. 7 (C–F)). It should be noted that both hydrogel beads exhibit higher ΔE values after exposure to DMA compared to TMA, especially at higher concentrations. A possible explanation for this observation can be related to pK_a_ values and nucleophilic attack of TMA and DMA to flavylium cation on anthocyanins structure. The pK_a_ value of DMA is 10.75, while it is 9.80 for TMA, indicating that DMA is a stronger base and more alkaline than TMA. Furthermore, the steric hindrance of TMA and DMA can influence their attack on positively charged flavylium cations. TMA has three methyl groups and a larger molecular size, which can hinder its access to the reactive sites on anthocyanins. Therefore, DMA is more likely to act as a nucleophile, meaning it can react more readily with anthocyanins than TMA (Luo and Lim, 2020). In both indicators, the LOD and LOQ for DMA and TMA were nearly identical (Table 5), which can be due to the similar ΔE values in the linear range of the curve, particularly up until 20 ppm. A comparison of the performance of two hydrogel beads in response to TMA revealed a significant difference (p< 0.05) in concentrations ≥1000 ppm, with the F_1_ presenting greater sensitivity than F_2_. On the contrary, F_2_ was more sensitive to DMA in the same concentration range.

During post-mortem storage, TMAO undergoes degradation through both non-enzymatic and enzymatic pathways, leading to the formation of TMA, DMA, and formaldehyde, which contribute to the deterioration of frozen and chilled fish products. A small amount of DMA, but mainly TMA, is generated via bacterial TMAO-reductase (Summers et al., 2017). It can be postulated that species with higher concentrations of TMAO will produce more TMA during storage. Deep-water fish species have been found to contain a higher TMAO level than shallow-water species (Summers et al., 2017). According to Etienne and Ifremer (2005), the species of fish with the highest concentrations of TMAO were dogfish (941–1170 mg/100 g) and skates (430–1830 mg/100 g). Hakes, gadoids, and redfish indicated intermediate levels of TMAO, ranging from 322 to 645 mg/100 g. The species of fish with the lowest levels of TMAO were typically pelagic and flatfishes (≤160 mg/100 g). Karpas et al. (2002) measured TMA levels in various muscle foods such as chicken, beef, turkey, and lamb after one day of storage at room temperature. It was reported that muscle food with high levels of fatty tissue, like pork, has elevated levels of TMA. Therefore, F_1_ hydrogel beads are ideal for developing intelligent packaging solutions for marine fish products or muscle foods with high-fat content.

It has been shown that DMA can be produced and used as a freshness indicator in gadoid species like cod, haddock, and whiting (Boziaris, 2014). Additionally, the production of DMA has been observed in smoked marine fish, including salmon, sable, and shad (Miller et al., 1972). Thus, integrating F_2_ hydrogel beads into the packaging of these products could be promising.

The Norwegian Food Safety Authority (Mattilsynet, 2019) sets a TMA limit of 3 mg/100g fresh lean fish species, herring, or mackerel. Moreover, fish or fishery products with TMA levels exceeding 10 mg/100g cannot be sold. The limit of detection for TMA in both hydrogel beads was 48 ppm corresponding to 4.8 mg/100g, showing the suitability of using both hydrogel beads in fishery product packaging.

## 4 Conclusion

In this study, hydrogel-based pH indicators were developed for intelligent packaging of muscle food products. The hydrogels of different sizes were produced by encapsulating the anthocyanins derived from purple corn powder within the alginate matrix using electrostatic extrusion. Smaller hydrogel beads demonstrated better stability than larger beads when subjected to accelerated light conditions or high RHs at 4 °C over an extended period, making them suitable for muscle food products with varying shelf lives. Large beads were only recommended in muscle food products with short shelf-life (no more than one week), considering stability. The use of hydrogel beads in the packaging of products with low relative humidity was found to be unsuitable due to issues such as excessive moisture loss and a darkened appearance. Thus, freeze-drying of hydrogels can be a potential alternative for future studies. The sensitivity of the hydrogel beads to specific amines varied. Large beads exhibited higher sensitivity to lower ammonia concentrations, making them well-suited for early-stage spoilage detection or products with lower levels of ammonia production. On the other hand, small beads were appropriate for products with higher levels of ammonia production. Both hydrogel beads showed similar limits of detection for dimethylamine and trimethylamine. However, significantly different performance was observed at concentrations exceeding 1000 ppm in a way that the small and large beads were more sensitive to trimethylamine and dimethylamine, respectively.

The results of this research provide significant insights into the practical application of these hydrogel bead indicators in different types of muscle food and highlight the importance of tailoring the choice of hydrogel bead based on specific product characteristics and the desired stage of spoilage detection. Overall, hydrogel beads developed in this study successfully exhibited desirable properties, such as color stability and responsiveness to volatile amines. However, the stability of anthocyanins plays a pivotal role in ensuring the long-term functionality of a pH indicator, and there is still room for further stability improvement through using fillers, antioxidants, or protective layers.

## CRediT authorship contribution statement

**Samira Mohammadalinejhad:** Conceptualization, Methodology, Formal analysis, Investigation, Visualization, Writing – original draft. **Marcin Kurek:** Supervision, Writing – review & editing. **Ida-Johanne Jensen:** Supervision, Writing – review & editing. **Jørgen Lerfall:** Conceptualization, Methodology, Supervision, Project administration, Writing – review & editing.

## Declaration of competing interest

I confirm that there are no known conflicts of interest associated with this manuscript and there has been no financial support for this work that could have influenced its outcome. I confirm that the manuscript has been read and approved by all named authors and that there are no other persons who satisfied the criteria for authorship but are not listed.

## Data Availability

Data will be made available on request.
