# Supplementary figures and images for: The potential of anthocyanin-loaded alginate hydrogel beads for intelligent packaging applications: Stability and sensitivity to volatile amines
Source: Curr Res Food Sci. 2023 Aug 5;7:100560. doi: 10.1016/j.crfs.2023.100560 (PMC10425905; doi:10.1016/j.crfs.2023.100560)

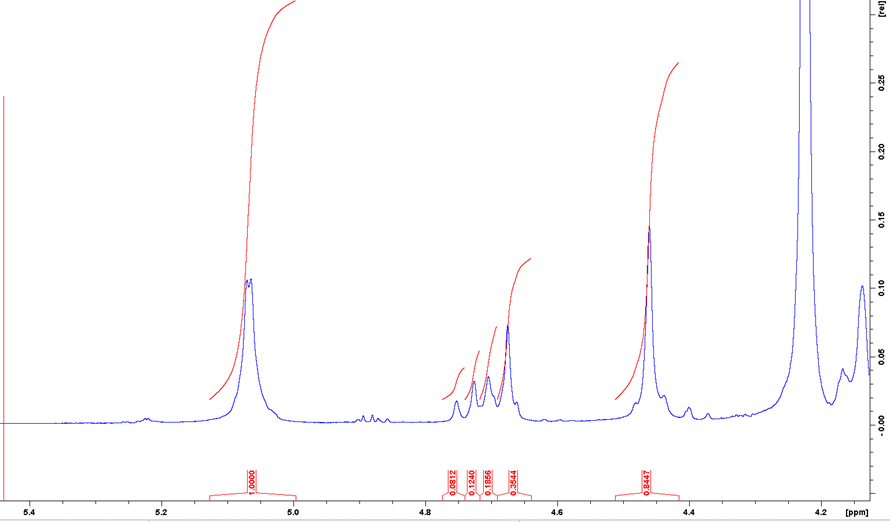


Fig S1. NMR spectrum of used alginate

Supplement: Multimedia component 1 [file mmc1.docx]
